# Real-World Study of Serum Neurofilament Light Chain Levels in Ocrelizumab-Treated People with Relapsing Multiple Sclerosis

**DOI:** 10.3390/jpm14070692

**Published:** 2024-06-27

**Authors:** Francisco J. Barrero Hernández, Ana Romero Villarrubia, Carmen Muñoz Fernández, Virginia Guillén Martinez, Almudena Aguilera Del Moral, José María Barrios-López, Maria A. Ramírez Rivas, Antonio J. Gálvez Muñoz, Raquel Piñar Morales

**Affiliations:** 1 Neurology Unit, University Hospital Clinic San Cecilio, 18016 Granada, Spain; ramirezrivasangelines@gmail.com (M.A.R.R.); rpinarmorales02@ugr.es (R.P.M.); 2 Instituto de Investigación Biosanitaria ibs.Granada, 18012 Granada, Spain; 3 Departament of Medicine, University of Granada, 18016 Granada, Spain; 4 Neurology Unit, University Hospital Virgen de las Nieves, 18014 Granada, Spain; aromerovillarrubia@hotmail.com (A.R.V.); vgm25m@gmail.com (V.G.M.); josemariabarrioslopez@gmail.com (J.M.B.-L.); 5 Neurology Unit, University Hospital Torrecárdenas, 04009 Almeria, Spain; mmunozf@outlook.es (C.M.F.); aaguileramo@gmail.com (A.A.D.M.); 6 Statistical Advisor and Methodology, Foundation for Biosanitary Research of Eastern Andalusia: FIBAO, 18016 Granada, Spain; ajgalvez@fibao.es

**Keywords:** disease-modifying treatment, multiple sclerosis, neurofilament, no evidence of disease activity, ocrelizumab, real world

## Abstract

Serum neurofilament light chain (sNfL) levels have been proposed as a biomarker of the clinical activity, disability progression, and response to treatment of people with multiple sclerosis (PwMS); however, questions remain about its implementation in clinical practice. Ocrelizumab (OCR) has proven effective in improving clinical and radiological outcomes and reducing sNfL levels. This real-life study followed the sNfL levels of 30 PwMS treated for 12 months with OCR and evaluated the usefulness of this biomarker for their short-term prognosis, considering expanded disability status scale (EDSS), annualized relapse rate (ARR), radiological activity, and NEDA-3 values. OCR reduced ARR in 83% of PwMS and radiological activity in 80%. EDSS was maintained, while NEDA-3 was achieved in 70% at 12 months. OCR produced an early reduction in sNfL levels (at 3 months). At baseline, greater MRI-evaluated radiological activity was associated with higher sNfL levels. sNfL levels over the first 12 months of treatment did not predict a suboptimal response or sustained control of the disease. Longer-term studies are needed to explore the predictive usefulness of sNfL levels in PwMS treated with high-efficacy drugs.

## 1. Introduction

Multiple sclerosis (MS) is a neurodegenerative demyelinating inflammatory disease related to axonal destruction, which is the most influential factor in the disability of people with MS (PwMS). This axonal neurodegeneration process releases proteins that form part of the cytoskeleton, such as neurofilament light chain in the cerebrospinal fluid (CSF) and serum [1]. Among biomarkers under investigation for the implementation of personalized medicine [2], serum neurofilament light chain (sNfL) is a promising candidate for assessing the activity, progression, prognosis, and response to treatment of PwMS [3]. Baseline sNfL levels have also been proposed as a potential predictive biomarker of disability in clinically isolated demyelinating syndrome [4].

CSF and serum neurofilament light chain levels correlate with each other [4], and their analysis is more accessible in serum by means of Single Molecule Array (SIMOA) technology [5]. Researchers are using SIMOA to investigate the relationship of sNfL levels with inflammation and neurodegeneration in PwMS [6], although no agreement has yet been reached on the sNfL levels associated with a worse prognosis. Elevated sNfL levels have been reported in PwMS with a greater frequency and severity of relapses [7] and in those with higher radiological activity [8], while they have also been associated with neurodegeneration and a greater risk of disability [9] and brain atrophy [10]. sNfL levels might therefore act as a predictive biomarker of clinical and radiological activity and of the response to different disease modifying treatments (DMTs), which is essential to determine suboptimal responses [11] and enhance the precision of MS therapies [12].

sNfLs are not MS-specific, also being elevated in active neuroaxonal lesions after traumatic brain injury, stroke, and other neurodegenerative diseases [13]. Physiologically, sNfL levels increase from the age of 60 years onwards, and reference values are given by age [14]. In a meta-analysis published by Cai et al., reported sNfL levels ranged between 9 and 35.9 pg/mL [15]. 

There is a need to establish sNfL cutoff levels by age group to differentiate between pathological and non-pathological conditions. Accordingly, a z-score was calculated, based on 10,133 samples from healthy individuals and adjusting sNfL levels by age and body mass index (BMI), reporting an association between a sNfL z-score >1.5 (percentile ≥94%) and a greater risk of clinical or radiological disease activity and/or worsening of the expanded disability status scale (EDSS) score in the following year [16]. It has been concluded that neurofilament light chain levels are more closely associated with inflammatory activity than with progression [17].

Ocrelizumab (OCR) is a recombinant humanized monoclonal antibody that selectively acts on B lymphocytes with surface expression of CD20 [18]. OCR has been found to significantly reduce the clinical activity and partially limit the progression of disability in PwMS [19]. OCR was reported to reduce sNfL levels in progressive forms in the ORATORIO trial [20] and in relapse forms in the OPERA trial [21], while another pivotal study observed an association between sNfL levels at 48 weeks and the risk of disability progression up to 9 years later [20]. In the ORATORIO trial, a 10-fold increase in baseline sNfL levels in the control group was associated with greater risk of progression as assessed by the Nine Hole Peg Test (9-HPT) (HR = 2.33, *p* = 0.036) and Timed 25-Foot Walk Test (T25FWT) (HR = 5.35, *p* = 0.003). The significant reduction in sNfL, independently of inflammatory activity, indicates that OCR treatment can also limit neuroaxonal damage.

The Ocrelizumab Biomarker Outcome Evaluation (OBOE) study (NCT02688985) reported a 30.8% reduction in sNfL levels after 12 months of OCR treatment and a correlation between sNfL levels and the number of Gd+ active lesions and/or increased T2 lesions in brain magnetic resonance imaging (MRI) [22]. The reduction in sNfL levels obtained by OCR was also found to be independent of baseline clinical or radiological activity [23]. A study in PwMS found no significant difference in sNfL values between OCR and rituximab (RTX) treatments (18.32 vs. 18.28 pg/mL, respectively) [24].

Various studies of PwMS have described a reduction in sNfL levels with the start of DMTs, which have demonstrated moderate (teriflunomide (TFL) [25], dimethyl fumarate (DMF) [26], or (high natalizumab (NTZ) [27], fingolimod (FGL) [28], alemtuzumab (ALM) [29], cladribine (CLD) [30], OCR [20], or ofatumumab (OFT) [31]) effectiveness.

Delcoigne et al. studied sNfL levels in 1261 patients receiving ALM, DMF, FGL, NTZ, TFL, or RTX and observed that baseline sNfL concentrations were positively associated with relapses and EDSS score, with all DMTs achieving a reduction in sNfL levels, which was highest with ALM and lowest with TFL [32].

sNfL levels have been considered as a biomarker of disability progression. A study of 578 patients followed for seven years associated levels > 10 pg/mL with a greater risk of progression and, independently, with a greater risk of disability during the first six months. In a multicenter study based on Real-Life Experience, PwMS with sNfL levels > 8 pg/mL had a 2.8-fold greater risk of deterioration, 4-fold greater risk of new lesions in T2, and 3.3-fold greater risk of relapse within two years [33]. However, other authors found no increased disability progression in PwMS with elevated sNfL levels treated with highly effective drugs [34]. Pivotal clinical trials, extension studies, and real-life studies have shown that sNfL levels can be reduced by treatment with both moderately and highly effective DMTs [35]. However, most of this evidence has been based on selected cohorts, largely in clinical trials, and few data have been published on populational cohorts [36].

The possibility to determine sNfL in peripheral blood has resulted in some research on sNfL levels in the clinical setting over recent years. However, the availability of SIMOA technology has been limited to date, and further research is required to determine the sensitivity and specificity of sNfL as a biomarker and to establish reference values and standardized protocols [1]. The objectives of this real-life study were to determine sNfL levels in PwMS before starting OCR treatment (baseline) and every 3 months for 12 months; to evaluate the effectiveness of OCR; to assess the usefulness of sNfL levels as a biomarker for short-term prognosis; and to explore their relationship with other clinical-demographic variables.

## 2. Materials and Methods

This prospective observational study included 30 PwMS prescribed OCR between 2021 and 2022 in three public hospitals in Southern Spain (San Cecilio Clinical and Virgen de las Nieves University Hospitals in Granada and Torrecardenas University Hospital in Almeria). OCR was prescribed following the routine clinical protocol at the hospitals, and MS was diagnosed in accordance with the 2017 McDonald criteria [37]. Data were gathered on demographics (age, sex, BMI) and clinical variables (age at disease onset, time with disease, number of DMTs received, the most recent DMT before OCR, and the reason for prescribing OCR). The effectiveness of treatment was assessed by results obtained at baseline and 12 months for the EDSS [38], annualized relapse rate (ARR), T25FW [39], 9HPT [40], and brain MRI, considering T1-weighted images before and after contrast (gadolinium-Gd) administration and T2/fluid-attenuated inversion recovery (FLAIR) sequences. No evidence of active disease (NEDA-3), recorded at baseline and 12 months, was defined by the absence of clinical relapse and sustained disability worsening (i.e., 1.5-point increase from baseline EDSS of 0, 1-point increase from EDSS of 1.0–5.5, and 0.5-point increase from EDSS > 5.5), and the lack of MRI activity [41].

Venous blood sNfL levels were measured at baseline and at 3, 6, 9, and 12 months using SIMOA technology (Quanterix, Billerica, MA, USA) with an SR-X instrument (Quanterix, Lexington, MA, USA) and NF-light Advantage Kit (Quanterix, Billerica, MA, USA); these analyses were conducted by the Immunology Department of the Ramón y Cajal Hospital in Madrid (Spain). The post-OCR response of sNfL levels was evaluated by calculating absolute mean sNfL values at baseline (sNfL-b) and 12 months (sNFL-12 m) and considering the ratio (sNfL-12 m/sNfL-b) and relative change in sNfL levels. Levels were also expressed as percentiles, and z-scores were calculated according to age- and BMI-adjusted standard reference values [16], with a z-score of sNfL > 1.5 (percentile ≥ 94) being associated with a higher risk of disease activity.

SPSS 28.0 (IBM SPSS, Armonk, NY, USA) and R version 4.2.0 (R Foundation for Statistical Computing, Vienna, Austria) were used for data analyses. Median values with interquartile range (IQR) were calculated for continuous variables and non-parametric statistics were therefore applied, using the Mann–Whitney U test for unpaired data and the Wilcoxon signed-rank test for paired data. Spearman’s rank-order correlation coefficient was applied to determine monotonic relationships between sNfL and clinical or demographic variables. Frequencies and percentages were calculated for categorical variables and their association were tested through the chi-square or Fisher’s exact tests. Clinical outcomes and sNfL levels at each time point were analyzed by means of a mixed-effects linear regression model to account for dependent (time series) observations and within subject variations. Confounders were included in the regression analysis to account for their potential effects on the clinical outcomes. Best model fit and model comparisons were determined in a stepwise procedure through ANOVA and comparison of Akaike Information Criterion to select the most parsimonious model [42]. Response and predictor variables were transformed when necessary to solve linearity and heteroskedasticity issues. Categorical outcome variables were analyzed in a similar manner by fitting generalized mixed-effects logistic regressions. A critical value of *p* ≤ 0.05 was set as the threshold for statistical significance in all tests carried out.

Ethical considerations: This study was performed in accordance with Good Clinical Practice and the Helsinki Declaration and was approved by the biomedical research ethics committee of Andalusia. Informed consent was obtained from all study participants.

## 3. Results

This study included 30 PwMS with forms of relapsing–remitting MS (RRMS) who had received OCR treatment for at least 12 months. At the onset of OCR treatment (baseline), the mean age was 40.8 ± 10.5 years, the mean BMI was 24.5 ± 3.81 (*n* = 21), and 57% were female; male and female participants did not significantly differ in sNfL levels (16.9 vs. 9.46 pg/mL, respectively; *p* = 0.368).

### 3.1. Baseline Clinical Characteristics and Follow Up

Table 1 lists the baseline clinical characteristics of the PwMS before OCR treatment: seven (23.3%) were naïve, seven (23.3%) switched from fingolimod, four (13.3%) from natalizumab, four (13.3%) from cladribine, three (10%) from interferon-beta, two (6.7%) from dimethyl fumarate, two (6.7%) from alemtuzumab, and one (3.3%) from teriflunomide. The switch was due to a suboptimal response in 29 (96.7%), relapse in 11 (36.7%), and clinical/radiological activity in 18 (60%), and for safety reasons in 1 patient. The mean number of previous treatments was 1.53 ± 1.33: one previous DMT had been received by 47.8%, two by 21.8%, and three by 30.4%.

At the 12-month follow-up, EDSS worsening was observed in one participant (3.3%), relapse in seven (23.3%), and radiological activity (new lesions in T2, no Gd+ activity) in two (8.3%). NEDA-3 was recorded in 70.8% of participants, a reduced ARR in 83.9% (0.23 ± 0.42 at 12 months vs. 1.43 ± 1.01 at baseline, *p* < 0.01). The mean EDSS (2.98 ± 1.89) did not significantly differ vs. baseline (3.1 ± 1.69), while active radiological lesions were detected in two participants (8.3%) vs. ten (34.5%) at baseline, an 80% reduction.

Table 1: Annualized relapse rate (ARR), dominant (d), disease-modifying treatment (DMT), expanded disability status scale (EDSS), magnetic resonance imaging (MRI) month (m), Nine Hole Peg Test (9HPT), non-dominant (nd) ocrelizumab (OCR), people with multiple sclerosis (PwMS), seconds (s), standard deviation (SD), Timed 25-Foot Walk (T25FW), year (y).

### 3.2. sNfL Levels and Clinical Outcomes

**Figure 1:**
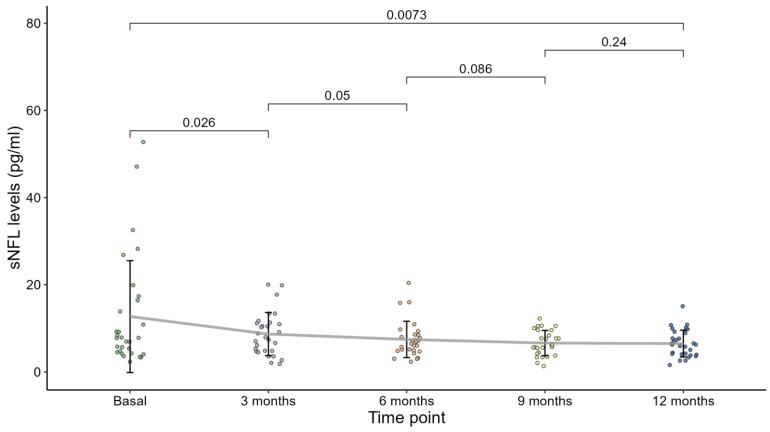
sNfL levels were compared among the different timepoints, using the non-parametric Wilcoxon test for paired samples to verify whether values in subsequent timepoints are lower, comparing “Baseline” with “3 months”, “3 months” with “6 months”, “6 months” with “9 months”, “9 months” with “12 months”, and “Baseline” with “12 months”. *p*-values of comparisons are indicated.

Figure 1 plots sNfL levels against measurement timepoints (baseline and 3, 6, 9, and 12 months). Each point represents an individual observation, with a line showing the trend of sNfL changes at each timepoint. Mean values with standard deviations were calculated for sNfL levels at each timepoint. The mean reduction in sNfL levels over 12 months was 6.21 ± 11.8 points, with a mean percentage sNfL reduction of 16.3 ± 55.5. The mean sNfL level was 12.7 ± 12.8 pg/mL at baseline vs. 6.48 ± 3.07 pg/mL at 12 months (*p* = 0.007), the mean z-score was 0.26 ± 1.99 at baseline vs. −0.615± 1.26 at 12 months (*p* = 0.008). At baseline, the sNfL level was >10 pg/mL in 33.3% at baseline versus 13.3% at 12 months, and the z-score was >1.5 in 40% at baseline vs. 3.3% at 12 months (*p* = 0.003). Among PwMS who had relapsed, 85.7% had shown an increase in sNfL levels during the three months before the relapse.

The median EDSS was higher in previously treated versus naïve patients (3.5 vs. 1.5, *p* = 0.002). In comparison to the former, naïve patients had more relapses during the previous year (2.43 ± 1.62 vs. 1.13 ± 0.46, *p* = 0.018) and higher radiological activity (mean of 1.43 ± 1.72 vs. 0.727 ± 1.70 Gd+ lesions, *p* = 0.047). At baseline, sNfL levels were similar between the groups (19.1 vs. 10.7 pg/mL; *p* = 0.174), although the naïve group had a higher mean sNfL z-score (1.55 ±1.5 vs. −0.134 ± 1.97, *p* = 0.033) and sNfL-b/sNfL-12 m ratio (*p* = 0.012).

The sample was divided between patients with and without NEDA-3 at 12 months to evaluate the influence of baseline sNfL levels on clinical and radiological outcomes. Table 2 exhibits demographic, clinical, and outcome data for the two groups. No significant between-group difference was found in baseline sNfL level (*p* = 0.710), baseline z-score (*p* = 0.26), sNfL level at 12 months (*p* = 0.153), z-score at 12 months (*p* = 0.852), reduction in sNfL (*p* = 0.114), percentage sNfL reduction (*p* = 0.065), or sNfL ratio (*p* = 0.065). A NEDA-3 binomial logistic regression analysis was independently performed with other variables such as age at onset of OCR, duration of the disease or EDSS without finding any statistical significance when controlling for these.

Table 2: Annualized relapse rate (ARR), dominant (d), disease-modifying treatment (DMT), expanded disability status scale (EDSS), magnetic resonance imaging (MRI) month (m), Nine Hole Peg Test (9HPT), non-dominant (nd) ocrelizumab (OCR), people with multiple sclerosis (PwMS), seconds (s) standard deviation (SD), Timed 25-Foot Walk (T25FW) year (y).

Likewise, no significant difference was found between patients with vs. without relapse during the 12-month follow-up in baseline sNfL levels (*p* = 0.441), baseline z-score (*p* = 0.532), sNfL levels at 12 months (*p* = 0.364), z-score at 12 months (*p* = 0.544), reduction in sNfL (*p* = 0.848), percentage sNfL reduction (*p* = 0.848), or sNfl ratio (*p* = 0.848).

Correlation analysis (Spearman’s rho) showed no relationship of baseline sNfL levels with age at baseline (−0.062; *p*= 0.746), age at disease onset (−0.045; *p* = 0.812), time with the disease (−0172; *p* = 0.364), relapses during the previous year (−0.086; *p* = 0.653), or EDSS score at baseline (−0.729; *p* = 0.136). A moderate correlation was found between baseline sNfL levels and radiological activity (Gd+ lesions) (0.458; *p* = 0.012).

No difference was found between patients with *versus* without radiological activity during the 12-month follow-up in baseline sNfL levels (*p* = 0.116), baseline z-score (*p* = 0.116), sNfL levels at 12 months (*p* = 0.958), z-score at 12 months (*p* = 0.587), reduction in sNfL (*p* = 0.116), percentage sNfL reduction (*p* = 0.087), or sNfL ratio (*p* = 0.087).

**Figure 2:**
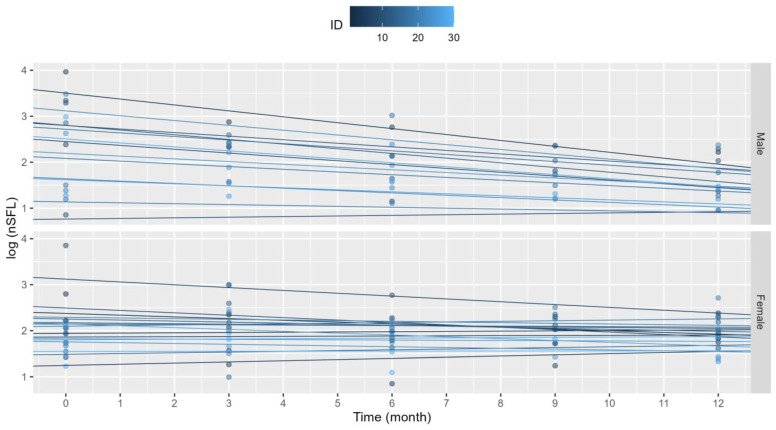
Mixed-effects linear regression models of each subject. OCR treatment appears to be more effective at reducing blood sNfL levels in males as shown by the steeper downward slopes of the fitted lines. Age at start of treatment also showed a significant reduction to treatment response on all subjects independently of sex.

Clinical outcome and confounding variables were analyzed in mixed linear regression models. The final model included the sex and age of participants at baseline. The adjusted model permits random intercepts and random slopes for each participant to improve the depiction of individual variability over time. OCR treatment showed to have a significant greater average reduction of sNfL levels in males compared to female participants. Age at start of treatment also showed a significant effect to OCR treatment, where older subjects showed worse response to treatment and lower reductions of biomarker levels overtime, in some cases even slightly increasing by the end timepoint (Figure 2). In the mixed effects binary logistic model on the most influential factors in relapses, the best fit was obtained for the sNfL levels at 3 and 9 months: with the odds ratio of a relapse rising to 0.386 per one-unit increase in sNfL at 3 months and to 2.035 per one-unit increase at 9 months.

## 4. Discussion

This real-world study followed PwMS treated with OCR for 12 months to determine the impact of this treatment on sNfL levels and other clinical, radiological, and disability outcomes, exploring the factors that influence this effect. Information provided by this type of study in a non-selected population complements data obtained in clinical trials [31,43].

In comparison to previous studies, the mean age at OCR onset was slightly higher (40 years), the time with disease was longer (around 12 years), the mean EDSS was lower, and the number of treatments was smaller (1.5 vs. 2.2), while ARR and Gd+ lesion load values were similar [33]. The main indication for this high-efficacy drug was a suboptimal response to moderate-efficacy (26% of participants) or high-efficacy (74%) drugs. ARR, disability progression (by EDSS, T25FW, and 9HPT), and MRI activity at 12 months were not associated with sNfL levels at baseline. The MRI finding of Gd+ lesions at OCR onset was moderately correlated with baseline sNfL levels (0.458. *p* = 0.012), in line with the study by Cross et al. [22]

NEDA-3 was reached in 70% of the PwMS, very similar to other results obtained using high-efficacy DMTs [30,44]. After 12 months of OCR treatment, the ARR was reduced in 83%, the EDSS was maintained, and there was an 80% reduction in those with radiological activity. In comparison, Sandgren et al. reported that 33% of ALM-treated patients reached NEDA-3 after a five-year follow-up, with a lower reduction in ARR [29].

Higher sNfL concentrations in CSF or serum have been associated with relapses, the emergence of new lesions in neuroimaging, and a worsening of the disease [45]. Clinical trials have determined sNfL levels in order to evaluate the effectiveness of different DMTs, but less information has been published on their usefulness for DMT follow-up in the routine clinical setting [16].

The significant reduction in sNfL levels verifies the molecular effect of OCR on neurodegeneration in PwMS and was observed at three months after the first infusion, earlier than reported for other DMTs [26]. The 16% decrease in sNfL levels at 12 months was lesser than described in the OBOE study with OCR [22] or in the study with CLD [30].

The clinical and/or radiological activity of this disease is associated with elevated sNfL levels and z-scores [46]. Interindividual differences in sNfL levels were minimized by comparing sNfL levels and z-scores [16], obtaining similar results for both. Besides the reduction in mean sNfL, the z-score was also decreased at 12 months vs. baseline, and the sNfL level reduction was maintained for at least 12 months. Most studies of PwMS have described a reduction in sNfL levels during the first 12 months, being greater and earlier with high-efficacy treatments (anti CD20, CD52, and integrin α4β1 antibodies) than with other oral therapies (S1P-receptor inhibitors, DMF, and TFL) or “platform” drugs [26,47].

Baseline sNfL levels were not associated with EDSS-assessed worsening, in agreement with previous findings [48]. sNfL levels in PwMS have been reported to rise during relapses and peak at three weeks [17]. In the present study, sNfL levels increased over the three months before relapse in 85.7% of the PwMS with relapses, compared with 38% of those treated with ALM [49]. As noted by other authors [5,50,51], not all PwMS with clinical “relapse” have a higher sNfL level than determined in the three previous months. Indeed, sNfL levels were not increased before the relapse in one out of six patients in the present population.

Unlike in another study [52], no association was observed between increased sNfL levels and greater disability assessed by EDSS, T25FW, or 9HPT test scores or between sNfL levels or baseline z-score and NEDA-3 at 12 months. This difference may be explained by the shorter follow-up time and/or the administration of a high-efficacy drug (OCR), given that Meier and coworkers found no association between sNfL levels and progression worsening in patients treated with anti-CD20 [53]. A real-life study of 14 cladribine-treated PwMS also found no relationship between sNfL levels and relapse, neuroimaging activity, EDSS progression, or NEDA-3 status [30]. Likewise, no correlation was reported between sNfL levels and NEDA-3 in 74 PwMS treated with CLD [54] or in 52 PwMS treated with DMF after 12 months of follow up [26]. However, studies with longer follow-up periods have demonstrated an association between baseline sNfL levels and the presence of clinical and radiological activity or EDSS progression [9,30,33,55]. Other authors have reported a modest association between sNfL levels and the degree of disability but not the frequency of relapses [36]. After a 12-year follow-up period, the only association observed by Canto et al. was between sNfL levels and EDSS scores [56]. Baseline ARR, EDSS, and radiological activity values were higher in naïve vs. previously treated patients, who had similar sNfL levels (19.1 vs. 10.7 pg/mL, respectively); however, the baseline level (1.55 vs. −0.134) and sNfL-b/sNfL-12 m ratio were higher in the naive group after adjustment by age and BMI (z-score). This may reflect the effect of DMTs on sNfL levels before the switch to OCR. Sandgren et al. also obtained higher sNfL levels in naive vs. previously treated PwMS group [29].

Study limitations include the modest sample size and follow-up period, preventing study of the mid- to long-term prognosis. In addition, it is more challenging to obtain short-term differences in sNfL levels and their relationship with relapses or disease progression in patients treated with a very highly effective drug such as OCR.

Despite the prognostic and monitoring value of serial determinations of sNfL levels, consensus groups recently concluded that these analyses will not replace MRI over the short term but will be used as a complementary technique or with a greater frequency, given their lower cost, thereby optimizing neuroimaging [57,58].

## 5. Conclusions

Given the unpredictable course and heterogeneous development of MS, biomarkers are needed to improve follow-up of the disease and the response to DMTs. OCR is a highly effective drug, as confirmed in this study, obtaining an ARR reduction in 83%, maintaining the EDSS score, and reducing radiological activity in 80% at 12 months of follow up, when 70% of the PwMS reached NEDA-3. The OCR treatment produced an early reduction in sNfL levels at three months that was maintained over the follow up period. Gd+ activity on the baseline MRI scan was associated with higher sNfL levels. The evaluation of sNfL levels during the first year of OCR treatment did not prove able to predict a suboptimal response or a persistence of disease control. The long-term evaluation of sNfL levels in wider studies is warranted to determine the predictive value of sNfL levels in PwMS treated with high-efficacy drugs.

## Figures and Tables

**Table 1:** Clinical–demographic characteristics before starting OCR and at one year of treatment.

	Before OCR	After One Year of OCR	*p* Value
MS onset age (y), mean (SD)	30.1 (9.9)		
Sex, female/male, *n* (%)	17/13 (56.5/43.3)		
Disease duration (y), mean (SD)	12.2 (9.39)		
Age (y), mean (SD)	40.8 (10.5)		
Time with OCR, (m), mean (SD)	24.3 (7.94)		
Baseline EDSS, median (range)	2.5 (1–6.5)	2.5 (1–7)	*p* = 0.072
ARR mean (SD)	1.43 (1)	0.23 (0.43)	*p* < 0.001
T25FW (*n* = 20) (s), mean (SD)	8.08 (5.78)	8.69 (7.5)	*p* = 0.649
9HPT-d (s) (*n* = 24) mean (SD)	29.2 (13.3)	29.4 (23.83)	*p* = 0.989
9HPT-nd (s) (*n* = 24) mean (SD)	29 (9.98)	28.30 (12.48)	*p* = 0.187
T2 lesion on MRI (*n* = 29), *n* (%)			
>20	19 (65.5)		
9–20	9 (31)		
<9	1 (3.4)		
T1 gadolinium enhancement. *n* (%)	10 (34.5%) (*n* = 29)	0 (*n* = 24)	*p* = 0.011
New or enlarged T2 lesions, (*n* =24) *n* (%)		2 (8.3%)	
PwMS with DMT before OCR, *n*, (%)	23(76.7)		
High-efficacy therapies	17 (73.9)		
Moderate-efficacy therapies	6 (26.1)		
PwMS naïve	7 (23.3)		

**Table 2:** Demographic, clinical, and evolution data of PwMS with or without NEDA-3 criteria.

	NEDA-3 (*n* = 17)	No NEDA-3 (*n*= 7)	*p* Value
MS onset age (y), mean (SD)	33.2 (10.6)	28 (8.08)	*p* = 0.294
Sex, F (%)	11 (64.7)	2 (28.6)	*p* = 0.182
Disease duration, (y) mean (SD)	11.9 (10.1)	2.71 (2.87)	*p* = 0.032
Onset age (y) OCR, mean (SD)	44.4 (9.47)	31.9 (8.51)	*p* = 0.006
Baseline EDSS median (range)	3.5 (1.0–6.5)	2 (1.0–3.5)	*p* = 0.044
ARR mean (SD)	1.24 (0.83)	2.14 (1.46)	*p* = 0.053
T25FW (*n* = 15) seg, mean (SD)	9.78 (7.47)	6.18 (0.62)	*p* = 0.489
9HPT-d (s) (*n* = 19) mean (SD)	32.3 (17.4)	27.3 (4.9)	*p* = 0.831
9HPT-nd (s) (*n* = 19) mean (SD)	32.3 (12.6)	28 (5.33)	*p* = 0.989
T2 lesion on MRI (*n* = 24), *n* (%)			*p* = 0.035
>20	13 (76.5)	2 (28.6)	
9–20	3 (17.6)	5 (71.4)	
<9	1 (5.9)	0	
T1 gadolinium enhancement. *n* (%)	5 (29.4)	3 (42.8)	*p* = 0.647
PwMS with DMTs before OCR, *n* (%)			*p* = 0.412
High-efficacy therapies	10 (58.8)	3 (42.85)	
Moderate-efficacy therapies	4 (23.5)	0	
PwMS naïve	3 (17.6)	4 (57.15)	

## Data Availability

Data is not available due to ethical or privacy restrictions. For any clarification you can contact the corresponding author.
